# Functional MRI-specific alterations in frontoparietal network in mild cognitive impairment: an ALE meta-analysis

**DOI:** 10.3389/fnagi.2023.1165908

**Published:** 2023-06-28

**Authors:** Xinyi Yang, Huimin Wu, Yu Song, Shanshan Chen, Honglin Ge, Zheng Yan, Qianqian Yuan, Xuhong Liang, Xingjian Lin, Jiu Chen

**Affiliations:** ^1^Department of Neurology, The Affiliated Brain Hospital of Nanjing Medical University, Nanjing, China; ^2^Department of Neurosurgery, The Affiliated Brain Hospital of Nanjing Medical University, Nanjing, China; ^3^Department of Radiology, The Affiliated Brain Hospital of Nanjing Medical University, Nanjing, China; ^4^Department of Radiology, Affiliated Drum Tower Hospital, Medical School of Nanjing University, Nanjing, China; ^5^Institute of Medical Imaging and Artificial Intelligence, Nanjing University, Nanjing, China; ^6^Medical Imaging Center, Affiliated Drum Tower Hospital, Medical School of Nanjing University, Nanjing, China

**Keywords:** activation likelihood estimation, amplitude of low-frequency fluctuation, frontoparietal network, functional connectivity, mild cognitive impairment, regional homogeneity

## Abstract

**Background:**

Mild cognitive impairment (MCI) depicts a transitory phase between healthy elderly and the onset of Alzheimer's disease (AD) with worsening cognitive impairment. Some functional MRI (fMRI) research indicated that the frontoparietal network (FPN) could be an essential part of the pathophysiological mechanism of MCI. However, damaged FPN regions were not consistently reported, especially their interactions with other brain networks. We assessed the fMRI-specific anomalies of the FPN in MCI by analyzing brain regions with functional alterations.

**Methods:**

PubMed, Embase, and Web of Science were searched to screen neuroimaging studies exploring brain function alterations in the FPN in MCI using fMRI-related indexes, including the amplitude of low-frequency fluctuation, regional homogeneity, and functional connectivity. We integrated distinctive coordinates by activating likelihood estimation, visualizing abnormal functional regions, and concluding functional alterations of the FPN.

**Results:**

We selected 29 studies and found specific changes in some brain regions of the FPN. These included the bilateral dorsolateral prefrontal cortex, insula, precuneus cortex, anterior cingulate cortex, inferior parietal lobule, middle temporal gyrus, superior frontal gyrus, and parahippocampal gyrus. Any abnormal alterations in these regions depicted interactions between the FPN and other networks.

**Conclusion:**

The study demonstrates specific fMRI neuroimaging alterations in brain regions of the FPN in MCI patients. This could provide a new perspective on identifying early-stage patients with targeted treatment programs.

**Systematic review registration:**

https://www.crd.york.ac.uk/prospero/display_record.php?ID=CRD42023432042, identifier: CRD42023432042.

## 1. Introduction

Mild cognitive impairment (MCI) depicts a transitory stage between natural aging and the onset of Alzheimer's disease (AD), affecting 10–15% of people over 65 years (Anderson, 2019). Patients with MCI have significant impairment in the cognitive domain (Jongsiriyanyong and Limpawattana, 2018). Many studies have found that multiple networks affect cognition control, including its realization, stabilization, maintenance, updating, and interaction (Zhou et al., 2015; Marek and Dosenbach, 2018). Coordinating behavior rapidly, accurately, flexibly, and purposefully is driven by the frontoparietal network (FPN). It is a flexible node to support cognitive control activation and helps respond to outside feedback (Marek and Dosenbach, 2018). Studies have paid attention to the structural integrity of nerve and brain regions suffering functional alteration in MCI using the resting-state functional magnetic resonance imaging (rs-fMRI) (Khazaee et al., 2016). However, no consistent results concerning specific changes in functional imaging of the FPN in MCI could be found. Thus, it becomes challenging to accurately conduct research on the details of resting-state imaging of MCI (Soman et al., 2020). Therefore, we attempted to summarize the valuable abnormalities of the FPN in MCI after analyzing previous investigations and associated research studies.

Brain fMRI is mainly performed based on the principle of blood oxygenation level-dependent (BOLD) contrast enhancement. It has been widely used in diagnosing and predicting AD progression (Sheline et al., 2010; Cai et al., 2017; Li et al., 2017; Wu J. et al., 2022). BOLD-fMRI can detect subtle abnormalities in brain function before MCI patients progress into the AD stage (Vannini et al., 2007). Current research usually adopts the following three methods to evaluate functional FPN alterations: (1) the amplitude of low-frequency fluctuation (ALFF)/fractional amplitude of low-frequency fluctuation (fALFF), (2) regional homogeneity (ReHo), and (3) functional connectivity (FC). ALFF can measure the amplitude of spontaneous regional brain activity by determining the square root of the power spectrum in the low-frequency range. The fALFF method is more sensitive than ALFF (Yang et al., 2020). Functional synchrony of low-frequency fluctuations during resting is an effective index to recognize preclinical AD, MCI, and normal aging (Li et al., 2002). ReHo analytical method is a highly reliable whole-brain data-driven approach based on the calculation of Kendall's coefficient of concordance (Liu et al., 2010). We could use the index to measure the homogeneity of time-series alternations between adjoining voxels and analyze spontaneous brain activity (Min et al., 2019). Seed-based FC can reflect the temporal correlations of the BOLD signal within different brain regions (Zhang et al., 2017).

The FPN serves as an essential functional component in the normal activities of human brains. Many studies on the pathophysiology of AD, MCI, schizophrenia, etc., put effort into the metabolic, structural, and functional alterations in the FPN. During typical aging, FC within the FPN declines, while changes accelerate in AD. These intra-network alternations co-occur with inter-network connectivity. Thus, shifting in network architecture could be compensatory in MCI (Wang et al., 2015). Consistent findings on brain networks converge toward fMRI alterations, including the executive control network (Xu et al., 2020), the default mode network (DMN) (Yuan et al., 2021), and the salience network (SN) (Song et al., 2021). The FPN could form the basis of executive control functions and share a high degree of FC without considering functional network organization (Cole et al., 2010; Power et al., 2013). Thus, the FPN is a unique control network that flexibly alters and interacts with other functional brain networks. Some scholars currently presume that a ubiquitous disconnection syndrome that leads to functional damage in resting-state brain networks is one of the leading causes of AD (Yildirim and Soncu Büyükişcan, 2019). Thus, MCI is precisely the clinical transition period for AD. Therefore, we can explore the consistent results for characteristic changes in the FPN in patients with MCI while summarizing its interactions with other networks. This can provide precious neuroimaging signs in predicting the early deterioration from MCI to AD.

This meta-analysis mainly applied an activation likelihood estimation (ALE) technique. It is a practical method based on random-effects interference that controls sample size. The technique holistically treated activation points in neuroimaging studies and pooled 3-D coordinates within stereotactic space from a certain amount of similar studies. These studies were published relative to Talairach or Montreal Neurological Institute (MNI) space and were spatially renormalized to a single pattern plate (Laird et al., 2005). The technique helps avoid the early shortage of non-determinacy about the subsistent abnormal position (Hétu et al., 2013). A previous study used ALE to identify that regional abnormalities could be an early predictor or diagnostic biomarker for MCI. However, our meta-analysis was the first to assess and analyze significant functional alterations in the FPN among patients with MCI.

Our primary intention was to systematically analyze and summarize the characteristic functional changes of brain areas in the FPN. These include changes within the network and its interaction with other networks in MCI. The following conjectures were put forward (1) three commonly used fMRI indexes (i.e., ALFF/fALFF, ReHo, and FC) of the FPN would depict specific neuroimaging changes without being ignored in some specific brain regions, and (2) interactions between the FPN and other networks could also have notable saliency among MCI individuals. We would offer a more meaningful perspective on the pathophysiological mechanism of the FPN in MCI patients after summarizing and understanding the characteristic functional alterations. This could help better reconstruct the disease progression along those lines for follow-up research.

## 2. Materials and methods

### 2.1. Literature search and study selection

This meta-analysis was conducted based on the Preferred Reporting Items for Systematic Reviews and Meta-Analysis (PRISMA) statement and the recommended checklist.

### 2.2. Search strategy

We searched PubMed, Web of Science, and Embase for English-language research papers in peer-reviewed journals up until July 2022 to identify studies for inclusion in this meta-analysis. The following search terms were used: (1) (frontoparietal network) AND (“mild cognitive impairment” [MeSH]) AND (“functional magnetic resonance imaging” [MeSH]) AND (Resting state) AND [(functional connectivity) OR (FC)], (2) (“mild cognitive impairment” [MeSH]) AND (“functional magnetic resonance imaging” [MeSH]) AND (Resting state) AND [ (regional homogeneity) OR (ReHo)] OR (local consistency), (3) (“mild cognitive impairment” [MeSH]) AND (“functional magnetic resonance imaging” [MeSH]) AND (Resting state) AND [(amplitude of low-frequency fluctuations) OR (ALFF)], and (4) (“mild cognitive impairment” [MeSH]) AND (“functional magnetic resonance imaging” [MeSH]) AND (Resting state) AND [(fractional amplitude of low-frequency fluctuations) OR (fALFF)].

**Figure 1:**
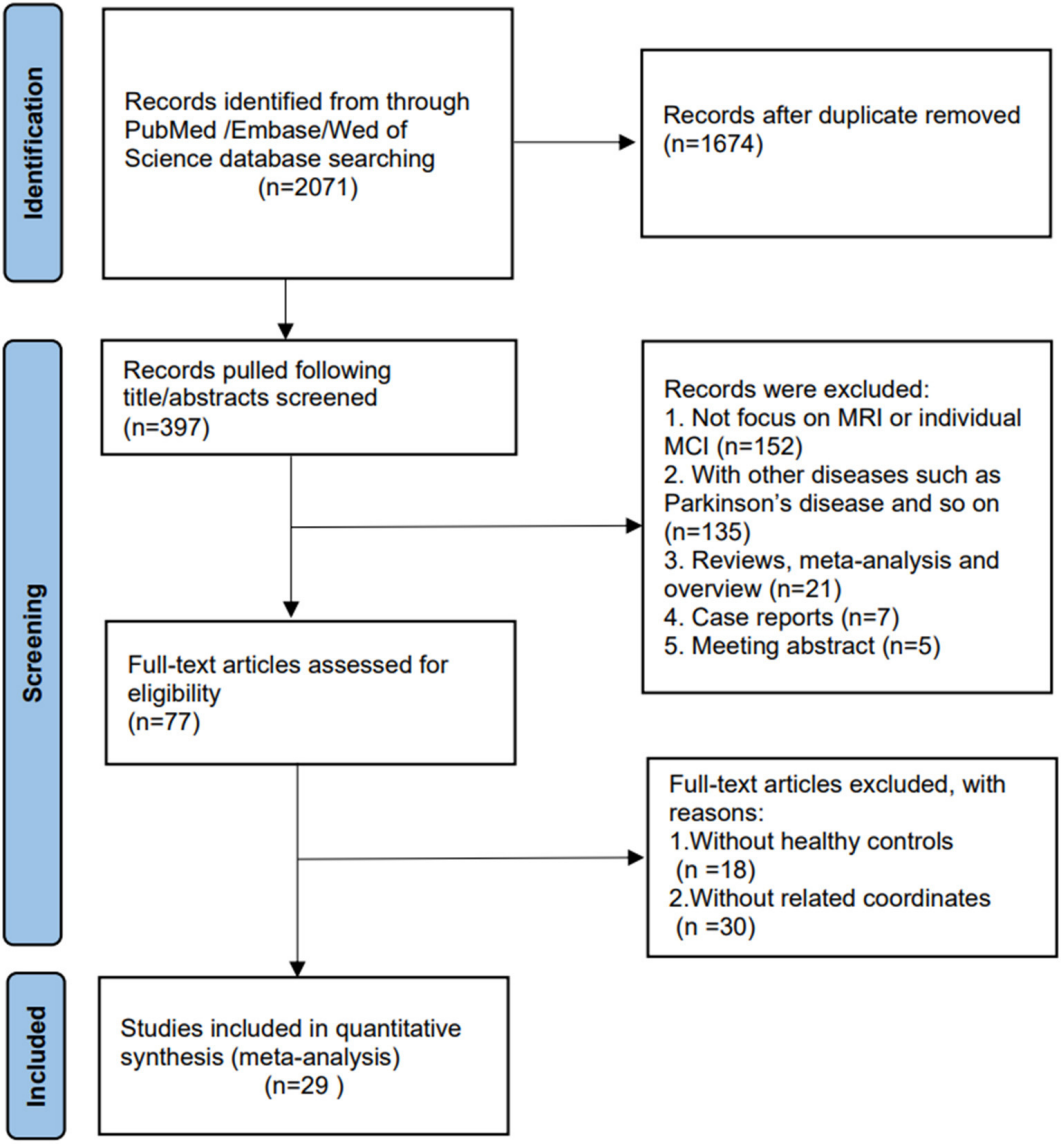
Flowchart showing the study selection process.

We initially selected 397 publications from the databases. After careful consideration, 29 publications (7 FC, 9 ALFF/fALFF, 12 ReHo, and 2 ALFF/fALFF & ReHo) were finally included in the analysis. We referred to the updated 2011 National Institute on Aging-Alzheimer's Association (NIA-AA) diagnostic guidelines for dementia (McKhann et al., 2011), MCI (Albert et al., 2011), and preclinical AD (Sperling et al., 2011). The selected publications were in the English language. The flowchart of the literature search and selection strategy is represented in Figure 1.

Many studies revealed rich individual diversity in the precise anatomical distribution of the FPN (Marek and Dosenbach, 2018). The brain areas of the FPN could be summarized as (1) Using seed ROIs: the lateral and medial portions of the dorsal and or the bilateral dorsolateral prefrontal cortex (DLPFC) (Marek and Dosenbach, 2018, 2019; Almdahl et al., 2021; Goodman et al., 2021; Matsuoka et al., 2021; Winters et al., 2021), the superior temporal gyrus (STG) (Marek and Dosenbach, 2018; Sharma et al., 2018; Goodman et al., 2021; Jandric et al., 2021), the angular gyrus (AG) (Marek and Dosenbach, 2018; Sharma et al., 2018; Jandric et al., 2021), the inferior parietal lobule (IPL) (Marek and Dosenbach, 2018; Goodman et al., 2021; Jandric et al., 2021; Matsuoka et al., 2021; Winters et al., 2021; Cui et al., 2022), the precuneus (ALFF) (Kaiser et al., 2015, 2019; Goodman et al., 2021), the anterior cingulate cortices (ACC) (Marek and Dosenbach, 2018; Goodman et al., 2021), the orbital part of left inferior frontal gyrus (IFG) (Jiang et al., 2020; Cui et al., 2022), the calcarine fissure (CAL) (Jandric et al., 2021; Cui et al., 2022), and the superior frontal gyrus (SFG) (Almdahl et al., 2021; Jandric et al., 2021; Winters et al., 2021); and (2) using independent component analysis (ICA): the orbital part of the inferior frontal gyrus (IFG), the middle temporal gyrus (MTG) (Cui et al., 2022), the right parahippocampal gyrus (PHG) (Marek and Dosenbach, 2019), the middle frontal gyrus (MFG), and the posterior cingulate cortices (PCC) (Goodman et al., 2021).

### 2.3. Data extraction and quality assessment

Two research team members comprehensively searched multiple databases, selected relevant literature, and extracted and grouped the data post cross-checks. Any disagreements were resolved by joint discussion.

Regarding studies concerning ALFF/fALFF and ReHo, the concrete selecting process is described. First, all the studies were reviewed, focusing on functional abnormalities in MCI and healthy controls (HCs), based on seed ROIs to summarize brain areas of the FPN. Then, the characteristic changes in brain regions of MCI patients were observed in imaging functional indicators, such as the ALFF/fALFF and ReHo. Finally, the aforementioned two groups of brain regions were sorted and compared, and the coordinates of the overlapping brain regions were extracted.

We selected those investigating the FC within the FPN or intra-FC between the FPN and other networks, such as the DMN. The coordinate of different brain regions was also highlighted, which showed substantial alterations.

### 2.4. Inclusion and exclusion criteria

We developed the following criteria for including articles: (1) clinical population of patients previously diagnosed with MCI or its subtype; (2) the selected subjects did not receive other additional interventions, including repetitive transcranial magnetic stimulation (rTMS); (3) parallel or cross-over design utilizing a healthy group or condition; (4) fMRI as a functional outcome measure; (5) information regarding Talairach or the MNI were indispensable; and (6) articles written in English. We initially screened studies identified through database searches based on their title and abstract. If their main research direction involved other diseases, such as schizophrenia, cognitive impairment due to other underlying disorders and Parkinson's disease, etc., they were eliminated. Studies were also excluded if they were irrelevant or did not meet the inclusion criteria from the abstract. If it remained unclear, the study was assessed in its entirety. We gave up literature that had been reprocessed, such as reviews and meta-analyses. Studies that lacked group-level statistics were also not included. Additionally, conference abstracts/papers were also excluded.

In addition, the Newcastle–Ottawa scale and a modified version for cross-sectional studies were utilized as the predefined data extraction sheets and quality assessment sheets (Stang, 2010). Scores of studies with 7–10, 5–6, and 0–4 points were, respectively, identified as high, moderate, and low quality (Arab et al., 2018). Of the included studies, 17 were of high quality, 12 were moderate, and none were of low quality. Screening, data extraction, and quality assessment were carried out by two authors (XY and YS), and disagreements were resolved by author consensus.

### 2.5. Data analysis procedures

The specific changes obtained by three different methods (ALFF/fALFF, ReHo, and FC) were systematically divided into two groups, such as increased and decreased: (1) decreased ALFF/fALFF (302 subjects, 16 foci, and eight experiments) and increased ALFF/fALFF (47 subjects, five foci, and three experiments); (2) decreased ReHo (434 subjects, 33 foci, and eight experiments) and increased ReHo (175 subjects, 15 foci, and seven experiments); and (3) decreased FC (193 subjects, 21 foci, and seven experiments) and increased FC (32 subjects, one focus, and one experiment).

The data calculation in this meta-analysis was primarily based on a Java-based version of Ginger ALE 2.3.6 (http://www.brainmap.org/ale) (Eickhoff et al., 2012). Many studies have confirmed that Ginger ALE plays a crucial role in determining whether there is anatomical or functional convergence of differences among complex coordinate-based human neuroimaging research (Mar, 2011). It helps us assess the coincident points of differences regarding the foci in studies. The extracted relevant data were sorted into six text files in the format recommended by the ALE guidebook, which uniformly converted Talairach coordinates into standard MNI ones (or vice versa). After importing foci information into the software, we used a text file to read it. Finally, we covered maps in the MNI 152 template and read them using the DPAB software (http://fmri.org/dpabi) (Eickhoff et al., 2012). Specific parameters were set: a threshold at *p* < 0.01 with the false discovery rate set to achieve the ALE map with a cluster-level family-wise error, with correction at *p* < 0.05 and 1,000 permutations.

## 3. Results

### 3.1. Search results

We summarized the demographics and characteristics of the included studies with concrete details in Table 1. Before formal adoption, a quality assessment was conducted for the 29 selected articles. The results are available in the Supplementary material.

**Table 1:** Demographic characteristics of the included rs-fMRI studies.

**Number**	**Study**	**Sample size (*n*)**	**Sex (m/f)**	**Age (years ±SD)**	**MMSE (SD)**	**Reference space**	**Group contrasts**	**Foci (*n*)**	**Threshold**
**ALFF/fALFF**									
1	Yang et al. (2020)	MCI 52	26/26	68.06 ± 9.32	24.52 ± 4.27	MNI	MCI > HC	0	*P < * 0.001 cor
		HC 55	22/23	63.41 ± 7.97	28.07 ± 2.14		MCI < HC	1	
2	Wang et al. (2019)	MCI 17	9/8	70.53 ± 4.54	24.47 ± 3.88	MNI	MCI > HC	0	*P < * 0.001 cor
		HC 16	8/8	68.56 ± 5.76	28.25 ± 1.39		MCI < HC	1	
3	Liu et al. (2022)	MCI 114	68/46	72.35 ± 5.23	24.11 ± 1.01	MNI	MCI > HC	0	*P < * 0.001 cor
		HC 101	65/36	71.69 ± 4.95	28.31 ± 0.97		MCI < HC	3	
4	Zhou et al. (2020)	MCI 24	10/14	69.8 ± 6.2	23.9 ± 3.6	MNI	MCI > HC	2	*P < * 0.05 cor
		HC 32	14/18	67.9 ± 6.4	28.0 ± 1.9		MCI < HC	1	
5	Xi et al. (2012)	MCI 18	8/10	67.39 ± 7.67	25.16 ± 3.43	MNI	MCI > HC	0	*P < * 0.001 cor
		HC 18	9/9	65.42 ± 5.75	28.14 ± 1.84		MCI < HC	3	
6	Wang L. et al. (2021)	MCI 50	27/23	65.840 ± 11.171	26.240 ± 0.960	MNI	MCI > HC	0	*P < * 0.005 cor
		HC 43	21/22	65.256 ± 9.691	29.093 ± 0.781		MCI < HC	2	
7	Wang et al. (2011)	MCI 16	7/9	69.38 ± 7.00	26.50 ± 1.03	MNI	MCI > HC	1	*P < * 0.05 cor
		HC 22	7/15	66.55 ± 7.67	28.59 ± 0.59		MCI < HC	4	
8	Jia et al. (2015)	MCI 7	2/6	74.1 ± 7.8	27.0 ± 2.3	MNI	MCI > HC	2	*P < * 0.01 cor
		HC 15	8/7	70.2 ± 7.1	29.2 ± 1.3		MCI < HC	0	
9	Zhuang et al. (2019)	MCI 11	7/4	71.91 ± 4.39	27.64 ± 1.36	MNI	MCI > HC	0	*P < * 0.005 cor
		HC 10	5/5	67.80 ± 2.39	27.90 ± 1.29		MCI < HC	1	
**ReHo**									
1	Bai et al. (2008)	MCI 20	10/10	71.3 ± 3.8	27.2 ± 1.6	MNI	MCI > HC	1	*P < * 0.005 cor
		HC 20	9/11	69.4 ± 3.8	28.3 ± 1.4		MCI < HC	3	
2	Min et al. (2019)	MCI 10	5/10	69.8 ± 2.658	25.90 ± 0.738	MNI	MCI > HC	0	P=0.006 cor
		HC 10	5/10	69.90 ± 2.601	29.30 ± 0.823		MCI < HC	2	
3	Wang et al. (2022)	MCI 28	4/13	68 ± 13	NA	MNI	MCI > HC	0	*P < * 0.005 cor
		HC 17	9/19	68 ± 10	NA		MCI < HC	1	
4	Liu et al. (2021)	MCI 28	14/14	68.39 ± 4.65	NA	MNI	MCI > HC	0	*P < * 0.05 cor
		HC 38	18/20	68.66 ± 5.09	NA		MCI < HC	3	
5	Liu et al. (2022)	MCI 114	68/46	72.35 ± 5.23	24.11 ± 1.01	MNI	MCI > HC	0	*P < * 0.001 cor
		HC 101	65/36	71.69 ± 4.95	28.31 ± 0.97		MCI < HC	4	
6	Zhang et al. (2020)	MCI 98	39/59	73.67 ± 6.78	28.42 ± 1.59	MNI	MCI > HC	0	*P < * 0.05 cor
		HC 64	37/27	75.75 ± 5.98	28.98 ± 1.37		MCI < HC	1	
7	Yuan et al. (2016)	MCI 36	19/17	66.8 ± 9.5	24.9 ± 3.4	MNI	MCI > HC	1	*P < * 0.001 cor
		HC 46	27/19	64.3 ± 7.8	28.5 ± 2.0		MCI < HC	4	
8	Wu Y. Q. et al. (2022)	MCI 12	6/6	64.33 ± 7.01	21.42 ± 4.56	MNI	MCI > HC	2	*P < * 0.01 cor
		HC 12	6/6	64.00 ± 6.18	27.83 ± 2.52		MCI < HC	2	
9	Zhang et al. (2021)	MCI 28	13/15	65.71 ± 6.895	NA	MNI	MCI > HC	1	*P < * 0.005 cor
		HC 37	15/22	63.86 ± 8.250	NA		MCI < HC	2	*P < * 0.001 cor
10	Zhang et al. (2012)	MCI 19	10/9	76 ± 8	27 ± 2	MNI	MCI > HC	6	*P < * 0.05 cor
		HC 21	12/9	70 ± 7	29 ± 1		MCI < HC	0	
11	(Liu et al. (2014)	MCI 12	1/11	59.3 ± 3.3	26.4 ± 0.9	MNI	MCI > HC	3	*P < * 0.01 cor
		HC 12	4/8	60.6 ± 5.8	29.8 ± 0.4		MCI < HC	2	
12	Zhang et al. (2010)	MCI 48	30/18	72.04 ± 4.42	NA	MNI	MCI > HC	1	*P < * 0.01c or
		HC 36	17/19	71.64 ± 3.72	NA		MCI < HC	9	
**FC**									
1	Zhang et al. (2017)	MCI 32	14/18	68.91 ± 6.76	26.66 ± 1.91	MNI	MCI > HC	1	P=0.01 cor
		HC 40	16/24	66.35 ± 4.83	28.35 ± 1.49		MCI < HC	1	P=0.02 cor
2	Tang et al. (2021)	MCI 25	9/16	66.92 ± 8.96	22.44 ± 2.86	MNI	MCI > HC	0	*P < * 0.05 cor
		HC 20	11/9	62.60 ± 6.95	27.15 ± 1.35		MCI < HC	4	
3	Zhou et al. (2015)	MCI 27	13/14	73.8 ± 7.8	26.8 ± 1.8	MNI	MCI > HC	0	*P < * 0.05 cor
		HC 27	16/11	69.2 ± 6.5	28.9 ± 1.0		MCI < HC	5	
4	Munro et al. (2015)	MCI 42	30/12	74.0 ± 8.4	27.4 ± 2.1	MNI	MCI > HC	0	*P* = 0.03 cor
		HC 42	30/12	NA	NA		MCI < HC	6	
5	Li et al. (2021)	MCI 26	8/18	64.93 ± 10.11	23.73 ± 3.85	MNI	MCI > HC	0	*P < * 0.05 cor
		HC 36	17/19	64.22 ± 6.97	28.13 ± 2.77		MCI < HC	1	
6	Binnewijzend et al. (2012)	MCI 23	15/8	71 ± 8	27 ± 3	MNI	MCI > HC	0	*P < * 0.005 cor
		HC 43	23/20	69 ± 7	29 ± 1		MCI < HC	3	
7	Pini et al. (2020)	MCI 18	14/4	74 ± 7	24 ± 3	MNI	MCI > HC	0	*P < * 0.005 cor
		HC 38	24/14	74 ± 5	29 ± 1		MCI < HC	1	

rs-fMRI, resting-state functional magnetic resonance imaging; MNI, Montreal Neurologic Institute; MCI, mild cognitive impairment; HCs, healthy controls; SD, standard deviation; ALFF/fALFF, the amplitude of low-frequency fluctuation/fractional amplitude of low-frequency fluctuation; ReHo, regional homogeneity; FC, functional connectivity; MMSE, Mini-mental state examination; ICA, independent component analysis; Seed ROI, seed regions of interest.

### 3.2. Meta-analysis results

**Figure 2:**
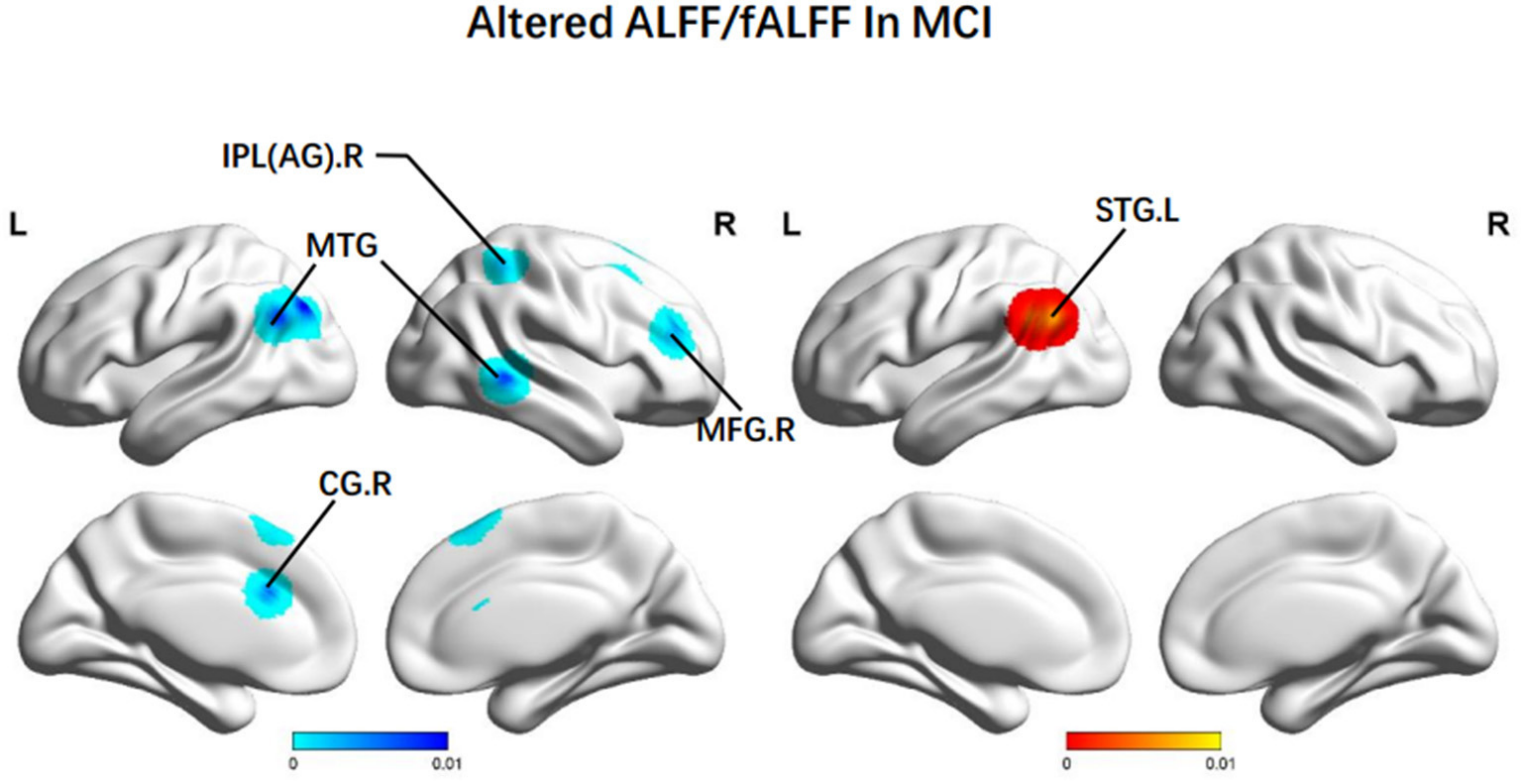
Brain regions showing altered ALFF/fALFF values in patients with MCI when compared with HCs. The color bar represents the p-value. Areas with decreased change relative to controls are displayed in blue, and areas with increased change are displayed in red. Results are thresholded at *p* < 0.01 and cluster-corrected, and a *p* < 0.05 family-wise error was corrected. MCI, mild cognitive impairment; HCs, healthy controls; ALFF/fALFF, the amplitude of low-frequency fluctuation/fractional amplitude of low-frequency fluctuation; IPL, Inferior parietal; AG, angular gyrus; MTG, middle temporal gyrus; MFG: middle frontal gyrus; CG, central gyrus; SFG, superior frontal gyrus; R, right; L, left.

Compared to HCs, patients with MCI showed completely different results in some brain regions:

In terms of ALFF/fALFF, patients with MCI presented higher scores in the left STG (BA 39). Decreasing ALFF/fALFF was identified in the right MFG (BA 9), the right MTG (BA 22), the left SFG (BA 6), the left AG (BA 40), and the bilateral CG (BA 24) (Figure 2).

**Figure 3:**
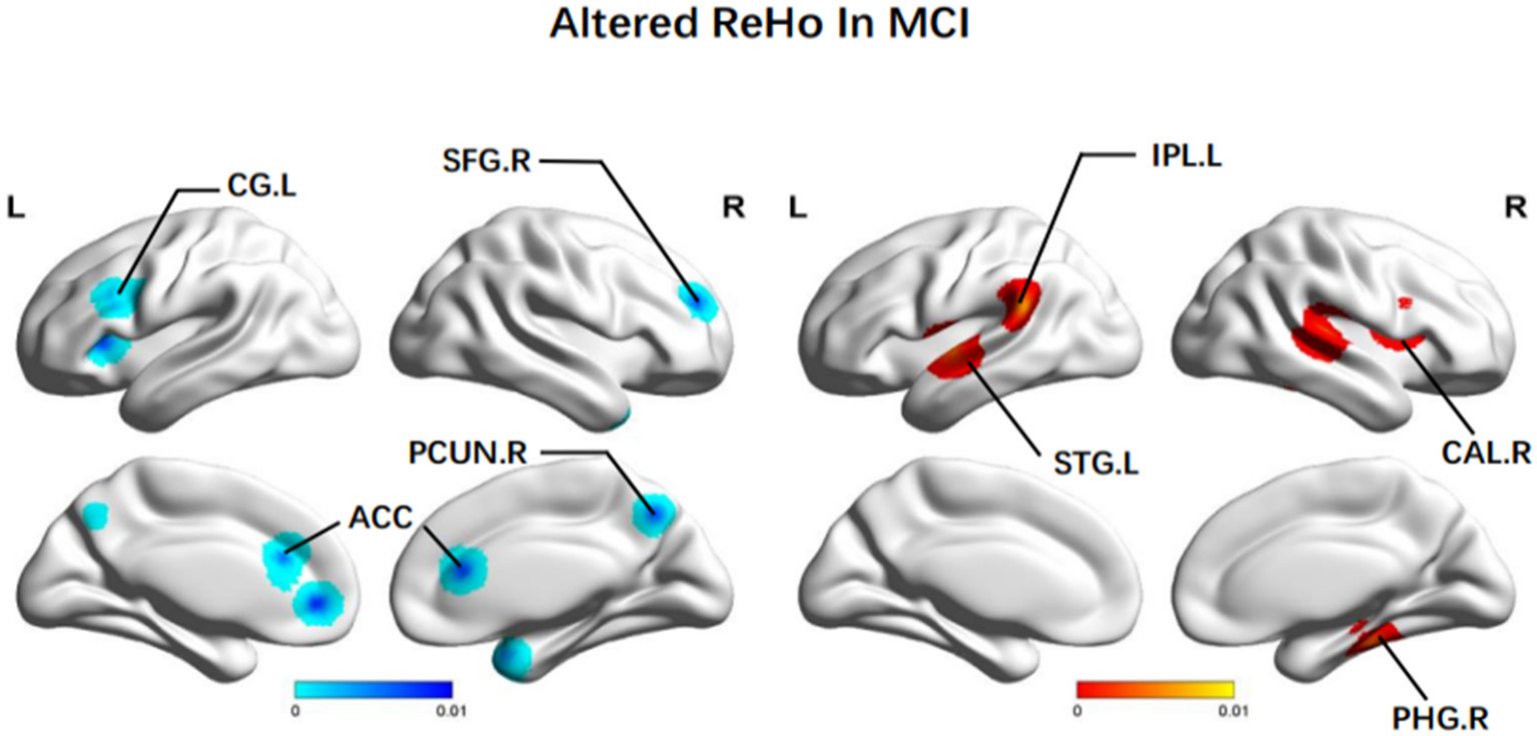
Brain regions showing altered ReHo values in patients with MCI when compared with HCs. The color bar represents the *p*-value. Areas with decreased change relative to controls are displayed in blue, and areas with increased change are displayed in red. Results are thresholded at *p* < 0.01 cluster-corrected and *p* < 0.05 family-wise error corrected. MCI, mild cognitive impairment; HCs, healthy controls; ReHo, regional homogeneity; SFG, superior frontal gyrus; CG, central gyrus; PCUN, Precuneus; ACC, anterior cingulate cortex; IPL, Inferior parietal; STG, superior temporal gyrus; CAL, calcarine fissure; PHG, parahippocampal gyrus; R, right; L, left.

In terms of ReHo, patients with MCI achieved increased ReHo in the right MTG (BA 41), the left STG (BA 22), the right PHG (BA 36), and the left IPL (BA 40). There was reduced ReHo in the right PCUN (BA 7), the left precentral gyrus (PreCG) (BA 6), the left CG (BA 32), the bilateral ACC (BA 32), and the left INS (BA 13) (Figure 3).

**Figure 4:**
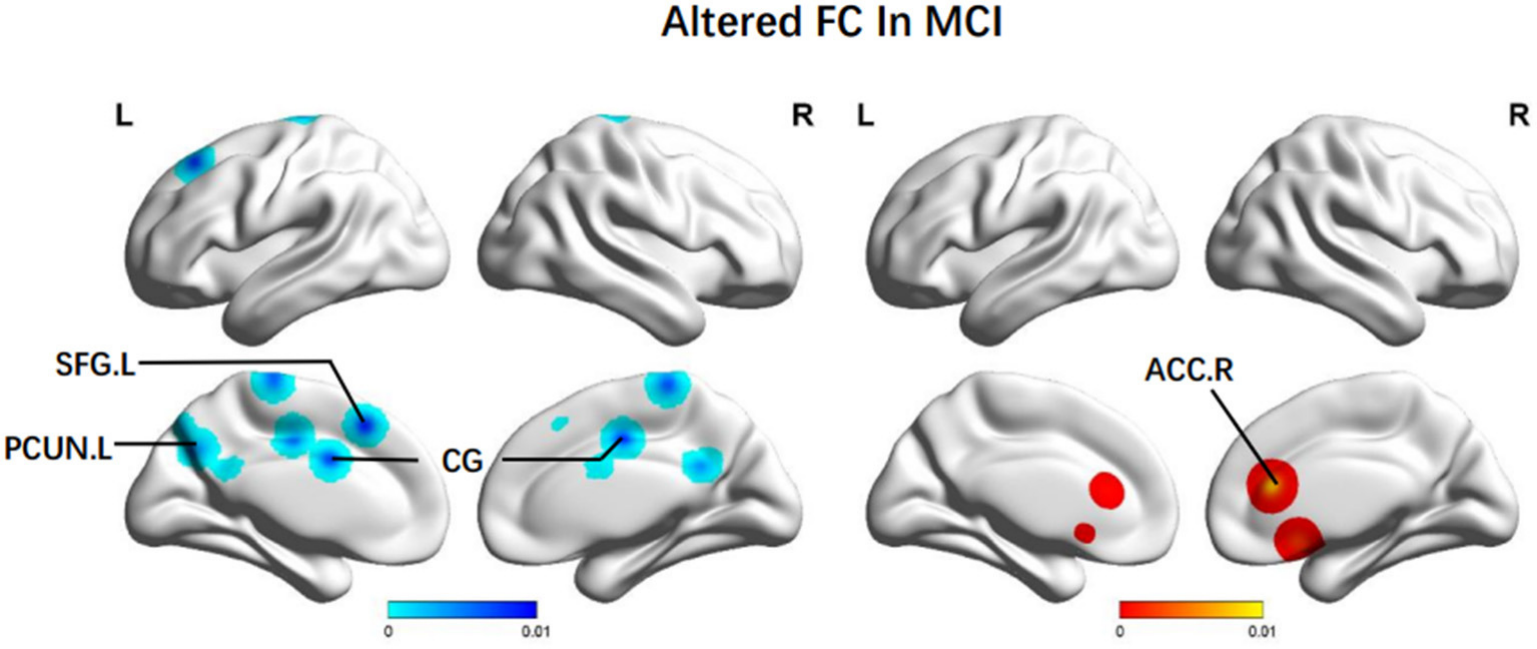
Brain regions showing altered FC values in patients with MCI when compared with HCs. The color bar represents the *p*-value. Areas with decreased change relative to controls are displayed in blue, and areas with increased change are displayed in red. Results are thresholded at *p* < 0.01 cluster-corrected and *p* < 0.05 family-wise error corrected. MCI, mild cognitive impairment; HCs, healthy controls; FC, functional connectivity; SFG, superior frontal gyrus; PCUN, Precuneus; CG, central gyrus; ACC, anterior cingulate cortex; R, right; L, left.

Additionally, brain regions presenting decreased FC were: the bilateral CG (BA 24, BA 31), the left PCUN (BA 7), the left paracentral lobule (BA 6), and the left SFG (BA 8). We also observed increased FC in the right ACC (BA 24) (Figure 4).

More details about clusters from the ALE analysis are demonstrated in Table 2.

**Table 2:** Regions with functional changes (ALFF, ReHo, and FC) between MCI and HC.

**Cluster**	**Volume(*mm*^3^)**	**MNI**			**Anatomical regions**	**Maximum ALE value**	**Side**	**BA**
		**X**	**Y**	**Z**				
**ALFF/fALFF**								
**MCI** > **HC**								
1	18,960	−54	−54	28	Superior temporal gyrus	0.00817	Left	39
1	18,960	−50	−63	29	Middle temporal gyrus	0.007665	Left	39
**MCI**<**HC**								
1	19,440	−42	−74	36	Middle temporal gyrus	0.010179	Left	39
1	19,440	−50	−64	30	Middle temporal gyrus	0.007669	Left	39
2	10,464	36	39	21	Middle frontal gyrus	0.007415	Right	9
3	10,016	60	−42	0	Middle temporal gyrus	0.00817	Right	22
4	9,488	0	26	60	Superior frontal gyrus	0.009786	Left	6
5	8,752	42	−45	57	Angular gyrus	0.007665	Left	40
6	8,448	−9	21	24	Cingulate gyrus	0.009733	Left	24
7	8,360	24	12	42	Cingulate gyrus	0.010494	Right	24
**ReHo**								
**MCI** > **HC**								
1	16,064	51	−27	9	Middle temporal gyrus	0.006546	Right	41
2	16,008	−54	−9	0	Superior temporal gyrus	0.006939	Left	22
3	12,080	36	−33	−24	Parahippocampal gyrus	0.008758	Right	36
4	10,008	−62	−44	23	Inferior parietal lobule	0.009139	Left	40
**MCI**<**HC**								
1	12,688	−50	18	28	Middle frontal gyrus	0.009466	Left	9
1	12,688	−40	4	28	Precentral gyrus	0.009373	Left	6
2	12,528	−2	30	24	Cingulate gyrus	0.010181	Left	32
2	12,528	6	30	16	Anterior cingulate	0.00818	Right	24
3	7,744	33	7	−31	Uncus	0.007624	Right	28
4	7,592	−36	24	4	Insula	0.008404	Left	13
5	7,296	3	−63	45	Precuneus	0.00818	Right	7
6	7,072	−8	45	−2	Anterior cingulate	0.009139	Left	32
7	7,056	29	49	25	Superior frontal gyrus	0.008519	Right	9
**FC**								
**MCI** > **HC**								
1	27,392	6	30	16	Anterior cingulate	0.00900	Right	24
**MCI**<**HC**								
1	13,112	−6	4	30	Cingulate gyrus	0.00934	Left	24
1	13,112	8	−8	40	Cingulate gyrus	0.008697	Right	24
1	13,112	−5	−15	42	Cingulate gyrus	0.008407	Left	24
2	12,904	2	−50	28	Cingulate gyrus	0.00958	Left	31
2	12,904	−4	−76	44	Precuneus	0.009341	Left	7
2	12,904	−12	−64	36	Precuneus	0.009066	Left	7
3	9,000	−0.5	−28.5	69	Paracentral lobule	0.000863	Left	6
4	8,640	−6	22	48	Superior frontal gyrus	0.009013	Left	8
4	8,640	−20	30	50	Superior frontal gyrus	0.007876	Left	8

BA, Brodmann Area; ALE, Anatomical/Activation Likelihood Estimation; MNI, Montreal Neurologic Institute; MCI, amnestic mild cognitive impairment; HCs, healthy controls; ALFF/fALFF, the amplitude of low-frequency fluctuation/fractional amplitude of low–frequency fluctuation; ReHo, regional homogeneity.

## 4. Discussion

Our meta-analysis is the first to assess the fMRI imaging alterations (ALFF/fALFF, ReHo, and FC) of the FPN in patients with MCI and healthy controls. The characteristic abnormal brain regions were primarily located in the bilateral MTG, the MFG.R, the IPL, the STG.L, the bilateral CG (ACC and PCC), the PCUN, the SFG, the CAL.R, the INS, the AG, and the PHG.R through professional data analysis and information integration.

The conclusion may become a favorable imaging predictor for early diagnosis of MCI or identification of high-risk individuals for AD by summarizing the characteristic changes in brain regions of the FPN in patients with MCI.

### 4.1. Abnormal imaging changes in the FPN

#### 4.1.1. Altered ALFF/fALFF in MCI

The MFG is primarily located in the lateral prefrontal cortex and makes up the core area of the DLPFC (Koenigs and Grafman, 2009). We attributed decreased ALFF of the MFG.R to the deteriorated executive functional performance among MCI groups. Among multi-domain MCI patients, major impaired cognitive domains are episodic memory, executive function, language, and visuospatial function (Wang S. et al., 2021). A previous study on the white matter changes within the uncinate fasciculus (UF) in patients with amnestic MCI (aMCI) described that the MFG is located in prefrontal brain regions closely connected with the UF. Thus, decreased ALFF values indicate the weakened spontaneous activity of neurons (Wang S. et al., 2021). It demonstrates that dysfunctional nerve cell activities and other structural changes in the FPN lead to executive dysfunction among patients with MCI.

Besides, ALFF values in the bilateral MTG were significantly lower than in the healthy controls (Min et al., 2019). It is believed that the MTG is a vital brain area associated with episodic memory (Budson and Price, 2005). Progressive episodic memory impairment is a prominent clinical symptom in MCI or AD patients, which could be connected to impaired medial temporal lobe function. Especially, the time-series homogeneity of BOLD signals in the medial temporal lobe system is significantly lower for a patient in the pre-AD period (Gómez-Isla et al., 1996; Dickerson et al., 2004; Greicius et al., 2004). Thus, this distinction can be used to build relevant predictive indicators and identify individuals at high risk of AD during MCI population screening.

The AG is an essential component of semantic processing, episodic stimulation, and episodic memory (Cavanna and Trimble, 2006; Thakral et al., 2017). The AG showing decreased ALFF from NC to MCI could indicate a decline of the neural vitality in the region as the common parts of the DMN and the FPN, as well as the key parietal nodes of the FPN. Then it could induce low efficiency of brain regions associated with controlling cognitive processes. Some correlation analyses indicated that the ALFF values of the AG, even in different bands, could be correlated positively with the MMSE score. With the development of MCI, the ALFF values of such patients in the regions mentioned above tend to decline along with their cognition level (Wang et al., 2022). Therefore, it was speculated that dysfunctional activities of nerve cells in the AG could be one of the leading causes of poor verbal working memory expression involving short-term storage and retrieval of phonological representations (Jonides et al., 1998; Bokde et al., 2010). Thus, using MMSE in the early screening of cognitive impairment indicates the possibility of constructing a dementia prediction model by combining simple mental scales and fMRI.

Furthermore, patients with MCI also had higher ALFF in the STG.L. It could be explained as compensatory redistribution or recruitment of cognitive resources. STG acts to identify and extract significative linguistic signals from speech inputs and is regulated by learning knowledge and perceived targets (Guo et al., 2017). A study related to low-frequency rTMS observed that the function of listening, comprehension, and speech in patients with aphasia could be elevated by inhibiting activities of the bilateral STG (Ren et al., 2019). This indicated that the increased activities in the STG.L could lead to a decline in language function among patients with MCI.

#### 4.1.2. Altered ReHo in MCI

Many studies revealed that the PCC/PCUN could have the highest metabolic rate and be seen as a tonically active brain region with high metabolic rates (Greicius et al., 2003). They play the role of gathering and extracting messages related to external and internal surroundings (Raichle et al., 2001). A study by Bai et al. (2008) reported that after statistically controlling for age, sex, and regional atrophy, ReHo was still declining over the PCC/PCUN. The results were consistent with previous rs-fMRI studies of MCI and further indicated that the decreased ReHo values could reflect the disrupted global cognitive function in patients with MCI (Li et al., 2002; Sorg et al., 2007). These alterations in fMRI imaging depicted that the changed PCC function could be due to the effects of persistent early neurodegeneration and low metabolism in the brains of patients with MCI. Moreover, it seems to be the most robust alteration in the MCI disease process.

Working memory is strongly dominated by the SFG, which is composed of multiple subregions. The SFG is also the core region of the DLPFC and serves as a key component in the FPN. Executive function loss in patients with MCI often occurs early in the disease and continues to progress. Consistent with our results, Zhang et al. (2021) reported reduced ReHo values of the SFG.R among aMCI patients. A study concerning the SFG cholinergic axon density in MCI and early-AD patients showed a selective up-regulation of the SFG in MCI. Thus, it presented unabiding and region-specific cholinergic neuroplastic reactions (Ikonomovic et al., 2007). Therefore, the SFG could be the major region consisting of various cognitive deficits in MCI, in structural and functional aspects (Liu et al., 2022). Besides, attenuated spontaneous neuronal activities of the SFG.R could identify MCI patients from HCs.

The bilateral ACC revealed significantly decreased ReHo in our fMRI neuroimaging research. The ACC is part of the limbic system of the brain and is integral to cognition, emotional processing, and executive function (Fillinger et al., 2018; Jung et al., 2019). Many previous neuroimaging studies have demonstrated that the ACC plays a prominent role in cognition and attention. Based on lesion studies, this region could be required for learning instrumental tasks and decision-making (Kolling et al., 2016; Aly-Mahmoud et al., 2017). Specific brain regions have a close relationship with MCI onset, and aMCI is linked with the dopamine-2 receptor binding in the ACC. Cognitive impairment involves the ACC even in its earliest prodromal form of subjective cognitive decline. Therefore, patients with MCI often have a decrease in the function of ACC in the early stage of the disease. Interestingly, proper physical activity can modulate the function and structure of the ACC, enhance the excitability of neurons, and significantly improve the cognitive level among patients with MCI (Tao et al., 2019).

Patients with MCI also displayed increased ReHo in some brain regions associated with memory and emotions, including the INS, the right CAL, and the right PHG. The INS is one of the most commonly impaired brain regions among patients with AD and is essential to cognitive function (memory, reason, language, executive function, etc.). A study performed at the acupoint KI3 in 12 patients with MCI revealed that ReHo increased in the INS during the pre-acupuncture resting state and became more pronounced following acupuncture (Laird et al., 2005). Greicius et al. (2004) suggested that IPL, CAL, and surrounding cortex processing are linked to increased regional homogeneity and memory (Sperling et al., 2003; Bokde et al., 2006; Dickerson et al., 2007). The higher regional homogeneity of MCI could compensate for the neurodegeneration of other memory-related regions. For example, this process involves the medial temporal regions and limbic system (Bai et al., 2008). Thus, based on neuropsychological information, memory function is only partially impaired in a specific subtype of patients with MCI.

Besides reduced intrinsic activity, higher ReHo in the left IPL was also observed in MCI. The IPL is vital in integrating messages from different sensory modalities and affects various higher cognitive functions (Caspers et al., 2006; Dickerson et al., 2009). Many studies observed that IPL is evoked in working memory and is wittingly or unintentionally buried within the recall of episodic memory information. Structural MRI studies have confirmed that IPL atrophy could predict aggravation from MCI to AD (Greene and Killiany, 2010). Our results were consistent with some previous findings (Qi et al., 2010; Xi et al., 2013). Increased functional changes of the IPL were a type of compensatory process. Patients with MCI could recruit network resources from the IPL to keep memory functions along with the declining MTL activity. This increased intrinsic activity may reflect episodic memory consolidation or retrieval elevation. The specific MCI-related compensatory mechanisms analyzed from the increasing IPL activations were also observed in task-fMRI studies during memory or mental processing (Yassa et al., 2008; Acosta-Cabronero et al., 2010; Bokde et al., 2010).

#### 4.1.3. Altered FC in MCI

Meanwhile, significant decreases of FC in MCI groups were observed in anterior-posterior brain connections, such as the SFG.L, temporal, and bilateral CG regions. Many researchers also found an anterior-posterior disconnection among AD and MCI patients (Sanz-Arigita et al., 2010; Neufang et al., 2011). This phenomenon could explain significant affective symptoms, for instance, apathy in dementia patients. Apathy is considered one of the highly prevalent symptoms of MCI and may be defined by the lowered resting-state FPN connectivity across different individuals. Several structural MRI studies have depicted the correlation between increasing white matter abnormalities and aggravated apathy in MCI. Thus, apathy-related disconnections between brain areas are prominent even in the initial phase of cognitive failure. The associations between more severe depressive symptoms and declined connectivity in healthy aging populations within the cognitive control network extensively overlap with the FPN (Alexopoulos et al., 2012; Yuen et al., 2014; Cheng et al., 2022). Our findings suggested that MCI individuals with more significant apathy symptoms lead to more defects in the network integrity of the FPN.

Even though the ACC and the PCC are adjacent and belong to the cingulate cortex together, FC results still had the opposite trend. As the anterior regions of recruiting cognitive information and initiating the message flow, the ACC had elevated FC. However, decreases in the PCC were appreciable in the group range, even at the earliest stage of symptomatic disease. A compensation mechanism could explain this to counter the decline of active nerve degree caused by posterior region impairments. Based on the findings above, aberrant spontaneous activities and gradual attenuation of neurons in regions associated with executive dysfunction and episodic memory can be observed in the MCI group. The brain activities of some regions were compensatorily increased, such as the ACC, to keep the liveness of the network and strengthen the connection between the front gyrus and medium/posterior brain regions (Jia et al., 2015). The hypo-connection could be the side effect of the low efficiency of the memory network, particularly in the temporoparietal region (Jacobs et al., 2012).

#### 4.1.4. Overlapped brain regions in the ALFF, ReHo, and FC

There is also significant overlap between different brain regions showing a decline in different indexes. These brain regions are mainly the PCUN, ACC, and SFG. These brain regions tend to have higher metabolic rates and are seen as more susceptible to early neurodegeneration. They play the role of gathering and extracting messages related to external and internal surroundings. These regions are connected with cognition, emotional processing, and executive function. They all perform a vital role in the formation of a certain kind of cognition until it successfully performs its function. Thus, we might attribute various cognitive deficits in patients with MCI to abnormal functional decline of brain regions mentioned above and that are involved with multi-dimensional cognitive function. This phenomenon also suggests that we can pay more attention to these active brain regions and try to use these brain regions as targets for individualized interventions or biomarkers for early monitoring of disease progression.

### 4.2. FPN interactions with other networks

Previous neuroimaging investigations have established that multiple brain functions are realized by intrinsically constructed brain networks, such as the FPN, the DMN, and the SN. These brain networks are essential in regulating sensory, motor, and even higher-level cognitive functions (Deco et al., 2011). Adjusting and controlling other networks could be potential features of the FPN, and vibrant interactions among networks highly correlate with cognitive dysfunction.

In terms of the ALE analysis results of the FC in the FPN, after adjusting for age, sex, and education level, the FPN interactions with the DMN and the anterior cingulate-insula network (AD) could be found in patients with MCI. The critical areas depicting interactions between the FPN and the anterior cingulate-insula network (aCIN) were predominantly distributed in the PCUN, the PCC, and the frontoparietal lobe region. They were essential brain regions of the DMN and the FPN. Moreover, the brain regions associated with the interactions between the FPN and the aCIN were the DLPC and the bilateral CG. Three brain networks have very different roles in mediating cognitive function. However, coincident anatomical areas, co-activation, or anti-correlated topological properties between networks may help resolve the individual functional parts of the brain areas in the FPN. Dysfunction in coordinating extensive brain functional networks could be discoverable in several brain diseases (Bassett and Bullmore, 2009; Pievani et al., 2011).

The interactions between the aCIN and the FPN could be explained by a relationship between driving and being driven. The ACC of the aCIN is mainly to monitor performance, signal the need for behavioral adaptation (Ridderinkhof et al., 2004), present intensive cognitive control, and implement behavioral adjustments in concert with DLPC (Ridderinkhof et al., 2004; Egner, 2009). Previous fMRI studies (Sridharan et al., 2008) have confirmed that the aCIN sends a dominant information flow to the FPN in goal-directed tasks. They trigger subsequent brain function activation, such as gathering visual, somatosensory, and auditory sensory inputs from the occipital, parietal, and temporal cortices. It also overlaps extensively with pre-motor/motor areas and attains support for motor outputs. Thus, the significant decline in FC of the bilateral CG (primarily the PCC), which is the common region between the aCIN and the FPN, could be responsible for the near memory decline, unresponsiveness and meaningless behavior among patients with early MCI. The interaction among networks could be a combined effect of anatomical distribution and structural location. A previous electrophysiological study observed the aCIN performs a Granger causal control to the FPN in behaviorally easier tasks (Chand and Dhamala, 2017). However, the FPN played Granger causal control to the aCIN in behaviorally harder tasks. This indicates that the interaction between the FPN and the aCIN is strongly associated with the activity state, and different task objectives could lead to distinct effects. This provides a reference for designing experimental protocols related to the inter-functional-effects across different brain networks.

Patients with MCI also showed disrupted connectivity in the DMN (Sorg et al., 2007; Zhou et al., 2008), including the SFG, the PCC, and the PCUN (Raichle et al., 2001; Greicius et al., 2003; McKiernan et al., 2003). Decreased FC of the DMN between the PCC and the ACC was non-negligible in patients with MCI. It was consistent with the early AD pathology, beginning from the MTG and involving the entorhinal cortex, PHG, and fusiform gyri (Du et al., 2004). The persistent connectivity breakdown of the PCC/PCUN could be proposed as a precursor of patients with MCI at high progression risk (Soman et al., 2020). When AD pathology progressed sufficiently to cause the clinical manifestations of MCI, tau protein emerged throughout the neocortex and caused extensive disconnection of FC in the PCC/PCUN (Luo et al., 2017). However, the connection between the cerebellum, the middle cingulate cortex, and ACC was enhanced and maintained until the dementia stage (Wang et al., 2006; Skouras et al., 2019). Thus, connectivity within and between extensive brain networks in a healthy aging population is modulated by regulating cognitive function in different regions. It could enhance the fault tolerance of the network to disease. The FPN is coupled with the DMN, and many studies identified that the topological properties of the DMN and FPN are anti-correlated. This comes from the effect of interactions between the DPLC of the FPN and the PCUN of the DMN (Long et al., 2016). Patients with MCI had a more significant clustering coefficient and a longer absolute path length than healthy control subjects (Goekoop et al., 2004). Therefore, the disruption in nodal connectivity strength within the FPN and the DMN spatially rests with regional distance. The FPN regions with altered connectivity strength corresponded to long-distance connections. In contrast, the DMN regions showing altered connectivity strength corresponded to short-distance connections, implying that distinct interactions between the FPN and the DMN could pause consciousness.

Recently, studies have shown great interest in the potential influence of the DMN and the FPN on dual-task performance and attempted to carry out a double-network model or two large-scale functional brain networks (Joo et al., 2016; Crockett et al., 2017; Li et al., 2019; Yu et al., 2019). Thus, more attention should be paid to comprehensively considering the interactions between different networks. Early establishment of a multi-network model would provide ideas for intervention treatment in the early stage, slowing the deterioration of patients with MCI.

## 5. Limitations

There are certain deficiencies in our meta-analysis. First, it fails to see the heterogeneity in different research including (1) the individual differences among the study subjects, (2) the different use of magnetic resonance models, (3) the pre-treatment steps change, and (4) the selection of different analytical methods. Second, we only looked at the MCI population without a more nuanced population breakdown. A meta-analysis based on the specific subtypes of MCI, including aMCI or the non-amnestic MCI, was not performed due to a lack of relevant documents. Third, the selected seed points of the FPN were significantly affected by the subjective will of the operator. This would affect the results presented in the end. Finally, we only adopted the literature written in English. Hence, the collected data could be incomplete.

## 6. Clinical implications

We made a quantitative neuroimaging analysis and depicted the neurobiological features of the FPN in patients with MCI based on previous studies to summarize the specific characteristics of the FPN in MCI and its subtype through functional fMRI imaging. Although there are certain deviations, unified conclusions can be drawn. It could help formulate a more individualized therapeutic schedule on moderate interventions such as drugs, acupuncture, transcranial direct current stimulation, rTMS, and task-related cognitive training. Meanwhile, altered brain regions of the FPN in rs-fMRI could be a trait marker to distinguish preclinical patients with MCI from healthy elderly, emphasizing the common imaging alterations in fMRI. These indicate that the PCC/PCUN, IPL.L, and bilateral ACC could be the non-negligible part of the pathophysiology of MCI. Moreover, they help better grasp the intrinsic neural mechanisms of MCI or AD. The results also offer precious insights into imaging methods for early diagnosis and effective interventions for MCI and AD.

## 7. Conclusions

We have observed the balancing mechanism between damage and compensation in the FPN of patients with MCI by identifying the functional alterations. Some brain regions, including the PCUN, the ACC, and the SFG, indicated a significant decrease in functional indicators. These altered brain regions are primarily involved in episodic memory and executive dysfunction, with regions prone to early deposition of tau protein in the FPN. Similarly, functional interactions with other networks in certain brain regions are also significant. These findings can distinguish patients with MCI in an early clinical phase by constructing an MCI prediction model associated with brain function imaging and offering targeted optimal treatment for such patients. It is also essential to have a lucid grasp of brain functions and identify more characteristic biomarkers for the predementia stage of AD spectrum disease. This could improve outcomes for patients with dementia, relieving the heavy mental pressure on families and decreasing the burden of medical care.

## Data availability statement

The original contributions presented in the study are included in the article/Supplementary material, further inquiries can be directed to the corresponding author.

## Author contributions

The study was designed by XY, HW, YS, SC, XLin, and JC. HG, ZY, QY, and XLia organized and downloaded the data. XY, HW, YS, and SC helped analyze the data and drafted the manuscript. XLin and JC modified the article and approved the submission. All the authors contributed to the article and approved the submitted version.
