# Supplementary material for: Functional MRI-specific alterations in frontoparietal network in mild cognitive impairment: an ALE meta-analysis
Source: Front Aging Neurosci. 2023 Jun 28;15:1165908. doi: 10.3389/fnagi.2023.1165908 (PMC10336325; doi:10.3389/fnagi.2023.1165908)
Supplement: Supplementary file 1 [file Data_Sheet_1.PDF]

Methodological quality assessment of the included case-control studies with the Newcastle Ottawa Scale								
Study	Selection				Comparability	Exposure			Scores
	Adequate definition of case	Representativeness of the case	Selection of controls	Definition of controls	Control for important factor	Ascertainment of exposure	Same method of ascertainment for cases and controls	No responserate	Total
[1]	★	★	★	★	★	★	★	-	7
[2]	★	★	-	★	★	★	★	-	6
[3]	★	★	-	★	★	★	★	-	6
[4]	★	★	★	★	★	★	★	★	8
[5]	★	★	★	★	★	-	★	-	6
[6]	-	★	-	★	★	★	★	-	5
[7]	★	★	★	★	★★	★	★	-	8
[8]	★	★	★	-	★	★	★	-	6
[9]	★	★	★	-	★	★	★	-	6
[10]	★	★	★	★	★★	★	★	-	8

[11]	★	★	★	★	★	★	★	-	7
[12]	★	★	-	★	★★	★	★	★	8
[13]	★	★	-	★	★	★	★	-	6
[14]	★	★	-	-	★	★	★	-	5
[15]	★	★	★	★	★★	★	★	-	8
[16]	★	★	-	★	★	★	★	-	6
[17]	★	★	★	★	★★	★	★	★	9
[18]	★	★	★	★	★★	★	★	-	8
[19]	★	★	★	★	★	★	★	-	7
[20]	★	★	★	★	★	★	★	★	8
[21]	★	★	★	★	★	★	★	-	7
[22]	★	★	-	★	★★	★	★	-	7
[23]	★	★	-	★	★★	★	★	-	7

[24]	★	★	-	-	★★	★	★	★	7
[25]	★	★	-	★	★	★	★	-	6
[26]	★	★	-	★	★	★	★	-	6
[27]	-	-	★	★	★	★	★	-	5
[28]	★	★	★	★	★	★	★	★	8
[29]	★	★	-	★	★★	★	★	★	8

★：met，-：unmet or not applicable

[1] Bai F, Zhang Z, Yu H, Shi Y, Yuan Y, Zhu W, Zhang X, Qian Y (2008) Default-mode network activity distinguishes amnestic type mild cognitive impairment from healthy aging: a combined structural and resting-state functional MRI study. *Neurosci Lett* **438**, 111-115.

[2] Min J, Zhou XX, Zhou F, Tan Y, Wang WD (2019) A study on changes of the resting-state brain function network in patients with amnestic mild cognitive impairment. *Braz J Med Biol Res* **52**, e8244.

[3] Jia B, Liu Z, Min B, Wang Z, Zhou A, Li Y, Qiao H, Jia J (2015) The Effects of Acupuncture at Real or Sham Acupoints on the Intrinsic Brain Activity in Mild Cognitive Impairment Patients. *Evid Based Complement Alternat Med* **2015**, 529675.

[4] Wang J, Yang C, Wei X, Zhang M, Dai M, Huang G, Huang W, Wen H, Dou Z (2022) Videofluoroscopic Swallowing Study Features and Resting-State Functional MRI Brain Activity for Assessing Swallowing Differences in Patients with Mild Cognitive Impairment and Risk of Dysphagia. *Dysphagia*.

[5] Zhuang L, Liu X, Shi Y, Liu X, Luo B (2019) Genetic Variants of PICALM rs541458 Modulate Brain Spontaneous Activity in Older Adults With Amnestic Mild Cognitive Impairment. *Front Neurol* **10**, 494.

[6] Liu L, Jiang H, Wang D, Zhao XF (2021) A study of regional homogeneity of resting-state Functional Magnetic Resonance Imaging in mild cognitive impairment. *Behav Brain Res* **402**, 113103.

[7] Yang L, Yan Y, Li Y, Hu X, Lu J, Chan P, Yan T, Han Y (2020) Frequency-dependent changes in fractional amplitude of low-frequency oscillations in Alzheimer's disease: a resting-state fMRI study. *Brain Imaging Behav* **14**, 2187-2201.

[8] Liu L, Wang T, Du X, Zhang X, Xue C, Ma Y, Wang D (2022) Concurrent Structural and Functional Patterns in Patients With Amnestic Mild Cognitive Impairment. *Front Aging Neurosci* **14**, 838161.

[9] Wang P, Li R, Liu B, Wang C, Huang Z, Dai R, Song B, Yuan X, Yu J, Li J (2019) Altered Static and Temporal Dynamic Amplitude of Low-Frequency Fluctuations in the Background Network During Working Memory States in Mild Cognitive Impairment. *Front Aging Neurosci* **11**, 152.

[10] Zhou QH, Wang K, Zhang XM, Wang L, Liu JH (2020) Differential Regional Brain Spontaneous Activity in Subgroups of Mild Cognitive Impairment. *Front Hum Neurosci* **14**, 2.

[11] Xi Q, Zhao X, Wang P, Guo Q, Jiang H, Cao X, He Y, Yan C (2012) Spontaneous brain activity in mild cognitive impairment revealed by amplitude of low-frequency fluctuation analysis: a resting-state fMRI study. *Radiol Med* **117**, 865-871.

[12] Zhang T, Luo X, Zeng Q, Fu Y, Li Z, Li K, Liu X, Huang P, Chen Y, Zhang M, Liu Z (2020) Effects of Smoking on Regional Homogeneity in Mild Cognitive Impairment: A Resting-State Functional MRI Study. *Front Aging Neurosci* **12**, 572732.

[13] Wang L, Feng Q, Wang M, Zhu T, Yu E, Niu J, Ge X, Mao D, Lv Y, Ding Z (2021) An Effective Brain Imaging Biomarker for AD and aMCI: ALFF in Slow-5 Frequency Band. *Curr Alzheimer Res*.

[14] Yuan X, Han Y, Wei Y, Xia M, Sheng C, Jia J, He Y (2016) Regional homogeneity changes in amnestic mild cognitive impairment patients. *Neurosci Lett* **629**, 1-8.

[15] Xi Q, Zhao XH, Wang PJ, Guo QH, He Y (2013) Abnormal intrinsic brain activity in amnestic mild cognitive impairment revealed by amplitude of low-frequency fluctuation: a resting-state functional magnetic resonance imaging study. *Chin Med J (Engl)* **126**, 2912-2917.

[16] Wu YQ, Wang YN, Zhang LJ, Liu LQ, Pan YC, Su T, Liao XL, Shu HY, Kang M, Ying P, Xu SH, Shao Y (2022) Regional Homogeneity in Patients With Mild Cognitive Impairment: A Resting-State Functional Magnetic Resonance Imaging Study. *Front Aging Neurosci* **14**, 877281.

[17] Zhang Z, Cui L, Huang Y, Chen Y, Li Y, Guo Q (2021) Changes of Regional Neural Activity Homogeneity in Preclinical Alzheimer's Disease: Compensation and Dysfunction. *Front Neurosci* **15**, 646414.

[18] Zhang Z, Liu Y, Jiang T, Zhou B, An N, Dai H, Wang P, Niu Y, Wang L, Zhang X (2012) Altered spontaneous activity in Alzheimer's disease and mild cognitive impairment revealed by Regional Homogeneity. *Neuroimage* **59**, 1429-1440.

[19] Liu Z, Wei W, Bai L, Dai R, You Y, Chen S, Tian J (2014) Exploring the patterns of acupuncture on mild cognitive impairment patients using regional homogeneity. *PLoS One* **9**, e99335.

[20] Zhang YW, Zhao ZL, Qi Z, Hu Y, Wang YS, Sheng C, Sun Y, Wang X, Jiang LL, Yan CG, Li K, Li HJ, Zuo XN (2017) Local-to-remote cortical connectivity in amnestic mild cognitive impairment. *Neurobiol Aging* **56**, 138-149.

[21] Binnewijzend MA, Schoonheim MM, Sanz-Arigita E, Wink AM, van der Flier WM, Tolboom N, Adriaanse SM, Damoiseaux JS, Scheltens P, van Berckel BN, Barkhof F (2012) Resting-state fMRI changes in Alzheimer's disease and mild cognitive impairment. *Neurobiol Aging* **33**, 2018-2028.

[22] Pini L, Wennberg A, Mitolo M, Meneghello F, Burgio F, Semenza C, Venneri A, Mantini D, Vallesi A (2020) Quality of sleep predicts increased frontoparietal network connectivity in patients with mild cognitive impairment. *Neurobiol Aging* **95**, 205-213.

[23] Li H, Gao S, Jia X, Jiang T, Li K (2021) Distinctive Alterations of Functional Connectivity Strength between Vascular and Amnestic Mild Cognitive Impairment. *Neural Plast* **2021**, 8812490.

[24] Soman SM, Raghavan S, Rajesh PG, Mohanan N, Thomas B, Kesavadas C, Menon RN (2020) Does resting state functional connectivity differ between mild cognitive impairment and early Alzheimer's dementia? *J Neurol Sci* **418**, 117093.

[25] Munro CE, Donovan NJ, Guercio BJ, Wigman SE, Schultz AP, Amariglio RE, Rentz DM, Johnson KA, Sperling RA, Marshall GA (2015) Neuropsychiatric Symptoms and Functional Connectivity in Mild Cognitive Impairment. *J Alzheimers Dis* **46**, 727-735.

[26] Tang F, Zhu D, Ma W, Yao Q, Li Q, Shi J (2021) Differences Changes in Cerebellar Functional Connectivity Between Mild Cognitive Impairment and Alzheimer's Disease: A Seed-Based Approach. *Front Neurol* **12**, 645171.

[27] Zhou B, Yao H, Wang P, Zhang Z, Zhan Y, Ma J, Xu K, Wang L, An N, Liu Y, Zhang X (2015) Aberrant Functional Connectivity Architecture in Alzheimer's Disease and Mild Cognitive Impairment: A Whole-Brain, Data-Driven Analysis. *Biomed Res Int* **2015**, 495375.

[28] Wang Z, Yan C, Zhao C, Qi Z, Zhou W, Lu J, He Y, Li K (2011) Spatial patterns of intrinsic brain activity in mild cognitive impairment and Alzheimer's disease: a resting-state functional MRI study. *Hum Brain Mapp* **32**, 1720-1740.

[29] Zhang Z, Deng L, Bai F, Shi Y, Yu H, Yuan Y, Wang K, Jiang T, Jia J, Zhang Z (2010) Alteration of resting brain function by genetic variation in angiotensin converting enzyme in amnestic-type mild cognitive impairment of Chinese Han. *Behav Brain Res* **208**, 619-625.
